# Simultaneous Quantification of Bone Edema/Adiposity and Structure in Inflamed Bone Using Chemical Shift‐Encoded MRI in Spondyloarthritis

**DOI:** 10.1002/mrm.26729

**Published:** 2017-06-06

**Authors:** Timothy J.P. Bray, Alan Bainbridge, Shonit Punwani, Yiannis Ioannou, Margaret A. Hall‐Craggs

**Affiliations:** ^1^ Centre for Medical Imaging University College London London United Kingdom; ^2^ Department of Medical Physics University College London Hospitals London United Kingdom; ^3^ Arthritis Research UK Centre for Adolescent Rheumatology University College London London United Kingdom.

**Keywords:** spondyloarthritis, chemical shift‐encoded MRI, inflammation, bone marrow

## Abstract

**Purpose:**

To evaluate proton density fat fraction (PDFF) and R2* as markers of bone marrow composition and structure in inflamed bone in patients with spondyloarthritis.

**Methods:**

Phantoms containing fat, water, and trabecular bone were constructed with proton density fat fraction (PDFF) and bone mineral density (BMD) values matching those expected in healthy bone marrow and disease states, and scanned using chemical shift‐encoded MRI (CSE‐MRI) at 3T. Measured PDFF and R2* values in phantoms were compared with reference FF and BMD values. Eight spondyloarthritis patients and 10 controls underwent CSE‐MRI of the sacroiliac joints. PDFF and R2* in areas of inflamed bone and fat metaplasia in patients were compared with normal bone marrow in controls.

**Results:**

In phantoms, PDFF measurements were accurate over the full range of PDFF and BMD values. R2* measurements were positively associated with BMD but also were influenced by variations in PDFF. In patients, PDFF was reduced in areas of inflammation and increased in fat metaplasia compared to normal marrow. R2* measurements were significantly reduced in areas of fat metaplasia.

**Conclusion:**

PDFF measurements reflect changes in marrow composition in areas of active inflammation and structural damage and could be used for disease monitoring in spondyloarthritis. R2* measurements may provide additional information bone mineral density but also are influenced by fat content. Magn Reson Med 79:1031–1042, 2018. © 2017 The Authors Magnetic Resonance in Medicine published by Wiley Periodicals, Inc. on behalf of International Society for Magnetic Resonance in Medicine. This is an open access article under the terms of the Creative Commons Attribution License, which permits use, distribution and reproduction in any medium, provided the original work is properly cited.

## INTRODUCTION

The spondyloarthritides are a group of inflammatory diseases involving the spine, lower limb joints, and entheses 1. Clinical features of spondyloarthritis include back pain, spinal stiffness and loss of mobility, which may be caused by inflammation, structural damage, or a combination of both 2, 3. Inflammation usually occurs in the form of spondylitis, spondylodiscitis, or spondyloarthritis. Sacroiliac joint inflammation—*sacroiliitis*—is a common feature. Structural manifestations include syndesmophyte formation and joint fusion, which are thought to be caused by chronic inflammation and subsequent new bone formation 1. However, bone destruction also occurs commonly and patients may suffer from generalized osteoporosis and increased fracture risk 4, 5, 6. Spondyloarthritis usually affects young people; patients often present in their 20s 1. An early age at disease onset is associated with a worse functional outcome 7. Patients presenting in childhood or adolescence have a particularly poor prognosis 8.

In recent years, there has been a move toward early identification and treatment of spondyloarthritis in an attempt to improve long‐term functional outcomes 9. Although biologic therapy has been shown to be effective at reducing the burden of inflammation, clinical assessment of response can be difficult, and it may be unclear if inflammation is adequately controlled 9. Furthermore, many patients undergo structural progression (particularly joint fusion) despite biologic therapy 10. As a result, MRI increasingly is used to monitor inflammation and structural damage 11, 12 and to facilitate therapeutic decision making 9, 13. Current clinical imaging protocols mainly rely on conventional spin echo sequences: fat‐suppressed T_2_‐weighted images are used to detect active inflammation (active inflammation causes an increase in bone marrow water content), whereas T_1_‐weighted images are used to evaluate structural features, including fat metaplasia (defined as an increase in bone marrow fat content that typically occurs in areas of previous inflammation) and joint fusion 14. However, existing imaging techniques have multiple limitations.

First, despite the fact that new bone formation and bone destruction both are key features of spondyloarthritis 4, spin echo sequences provide minimal information about trabecular structure or density. Arguably, this has contributed to the uncertainty around the nature of structural changes in the joint and adjacent bone marrow in chronic sacroiliitis 12, 15. For example, it has been argued that fat metaplasia is an intermediate stage in the bone‐forming pathway 15, but there are no existing data on whether trabecular bone density is altered in these areas. Similarly, it has been argued that the appearance of T1‐hyperintense tissue in the joint itself (*backfill*) is an indicator of new bone formation 15, but no measurements of bone mineral density have been performed in these areas.

Second, image assessment relies on visual comparison of differences in image intensity after acquisition by a radiologist or rheumatologist. This method is highly subjective and relies on multiple sequences, none of which are entirely specific for inflammation. For example, the quality of fat suppression using the short tau inversion recovery (STIR) sequence is critical for assessment of active inflammation, but in our experience this is variable and often suboptimal. In addition, scoring systems involve binary choices as to whether inflammation is present in a given joint quadrant, and further binary choices regarding the depth and intensity of inflammation 11. Assessment of edema intensity requires a somewhat arbitrary comparison with the brightness of presacral blood vessels 11, and therefore is dependent on the sequence parameters and the scanning platform. Accordingly, agreement between observers' assessment of inflammation is suboptimal even in a controlled research setting at a single site 16, 17, and could be poorer still in clinical practice. Finally, spin echo‐based methods are relatively slow, which makes them less well suited to whole body imaging. Whole body imaging may be useful for identifying occult joint inflammation 18, 19, but using STIR sequences is time‐consuming and therefore impractical for patients with stiff and painful joints.

As a result of these limitations, there is an unmet need for quantitative physical measurements of bone marrow characteristics in patients with spondyloarthritis. Chemical shift‐encoded MRI (CSE‐MRI) 20 can accurately measure bone marrow composition in terms of proton density fat fraction (PDFF) 21, and therefore could be used to assess marrow composition. Furthermore, the presence of trabeculae in cancellous bone increases the rate of signal decay R2* because bony trabeculae are more diamagnetic than the surrounding marrow 22. Thus, R2* may be regarded as a marker of trabecular bone mineral density (BMD). The rate of signal loss R2* is thought to be related to trabecular BMD in an approximately linear fashion 23, 24. However, R2* measurements may be biased by variations in fat content 25, and if unaccounted for, R2* decay may also confound the measurement of fat content 26.

In this study, we evaluated PDFF and R2* measurements as potential markers of bone marrow composition and structure in inflamed juxta‐articular bone. To assess the relationship between BMD and R2* in the presence of fat, and between known FFs and PDFF measurements in the presence of bone, we constructed a series of phantoms containing varying proportions of fat, water, and trabecular bone and then imaged these phantoms using CSE‐MRI. In vivo, we performed CSE‐MRI in the sacroiliac joints of patients with spondyloarthritis and examined whether there were significant differences in PDFF and R2* between areas of active inflammation, fat metaplasia, and normal marrow.

## THEORY

Using a gradient‐echo based CSE‐MRI acquisition, the signal *S*(*t*_*n*_) acquired at the nth echo time *t*_*n*_ can be written:

$$S\left(t_{n}\right)\;=\;\left(\rho_{W}+\rho_{F}\overset{M}{\underset{m=1}{\sum }}r_{m}\exp\left(i2\pi f_{F,m}t_{n}\right)\right)\;\text{exp}\left(i2\pi f_{B}t_{n}\right)\text{exp}\left(-t_{n}R_{2}^{*}\right)$$

where *ρ*_*W*_ and *ρ*_*F*_ are the amplitudes of water and fat components; *f*_F,m_ is the frequency of each spectral fat component; *r*_*m*_ is the relative amplitude of each spectral fat component; M is the total number of fat components; and R2* = R2 + R2′, where R2 and R2′ are the rate constants for irreversible and reversible spin dephasing, respectively. In cancellous bone, the presence of trabeculae predominantly increases R2′ 22, although R2 also may be increased due to diffusion of protons in gradients generated by susceptibility differences 22, 23. Previous studies suggest that the relationship between marrow BMD and R2* is approximately linear 23, 24. The presence of fat and noise can both potentially confound R2* measurements, although the use of a multipeak spectral fat model can help reduce this bias 25, 27.

For the purposes of fat quantification, R2* can be assumed to be equal for all fat and water components 26, 28 or can be modelled separately for fat and water. Although using separate R2* decay terms for water and fat is feasible in trabecular bone 29, it has been argued that the use of a single ‘system’ R2* term is more practical and results in greater robustness to noise, and also may be particularly suitable in short‐T2* environments 30. Previous phantom studies suggest that marrow fat quantification using a single R2* term is accurate 21. For these reasons, we have opted against using dual R2* terms in this work and have instead assumed a single marrow‐system R2*.

## METHODS

This study received ethical approval from the Queen Square Research Ethics Committee, London, United Kingdom (Research Ethics Committee reference 15/LO/1475). All participants gave written informed consent prior to study entry.

### Fat–Water Phantom

A fat–water phantom consisting of twelve 50‐mL centrifuge tubes with varying true fat volume percentages was constructed based on phantoms previously described 31, 32, with fat percentages chosen to reflect the range of FF values observed in both normal and pathological bone marrow. True fat volume percentages in the phantom were 0, 10, 20, 30, 40, 45, 50, 55, 60, 65, 70, and 100%. Peanut oil was used as a surrogate for human fat because its NMR spectrum is similar to that of human adipose tissue 26, 33 and comprises approximately 9% palmitic, 4% stearic, 55% oleic, and 27% linoleic acids 34. In this work, we adopt the approach of Gee et al. 21 and use fat volume percentages as reference FFs. For each tube, the appropriate volume of peanut oil was dispensed by weight, assuming the density of peanut oil (0.916 g/cm^3^). Sodium dodecyl sulphate (SDS) (surfactant; Sigma‐Aldrich, St. Louis, Missouri, USA) was added to the peanut oil and gently mixed to form an initial emulsion, ensuring a final SDS concentration in each phantom of 28 mM. Appropriate volumes of 3.0% weight/volume agar solution preheated to 90 °C were then added to each tube, and each tube was mixed by gentle inversion for approximately 2 minutes. The tubes cooled at room temperature, and all formed a solid gel (with the exception of the 100% FF tube, which contained pure oil).

**Figure 1:**
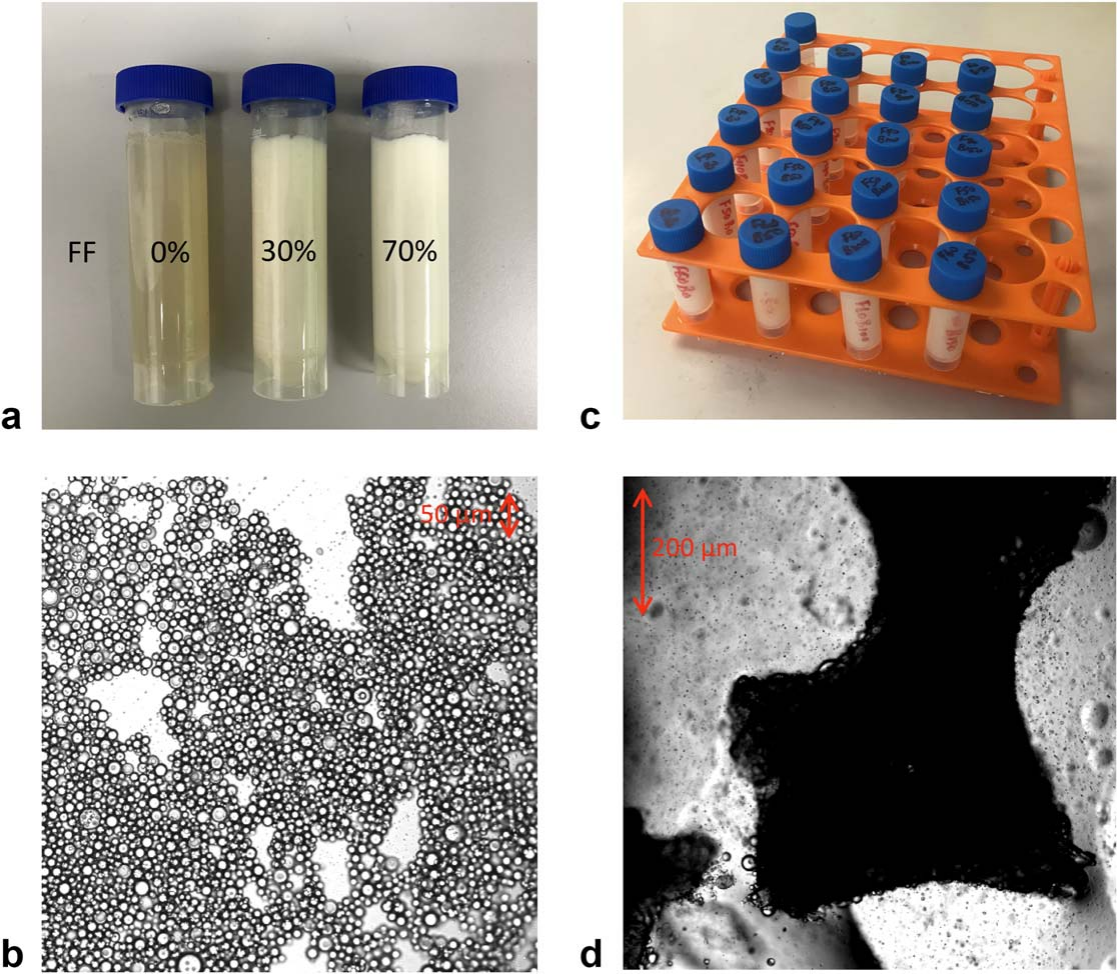
Fat–water (**a**, **b**) and fat–water–bone (**c**, **d**) phantoms. (**a**) 0%, 30%, and 70% FF vials are shown lying flat to demonstrate solidity. (**b**) Microscopic images of 50% FF tube shows the arrangement of fat and water in the emulsion (the fat globules are circular and outlined by dark lines). (**c**) Photograph of the fat–water–bone phantom, showing fat content varying by row and bone mineral density varying by column. (**d**) Microscopic images of trabecular bone granules in dilute emulsion (approximately 10% FF); a number of fat droplets are visible coating the surface of the trabeculae. FF, fat fraction.

Photographs of the 0%, 40% and 70% FF tubes from the fat–water phantom are shown in Figure 1a; microscopic images of a drop of the fresh emulsion (Zeiss LSM 710 confocal microscope; Carl Zeiss, Oberkochen, Germany) are shown in Figure 1b.

### Fat–Water–Bone Phantom

The fat–water–bone phantom consisted of fat–water mixtures with the addition of granules of decellularized bovine bone matrix (NuOss granules, particle size 250–1,000 μm, Henry Schein, London, UK), which are physically and chemically similar to the mineral matrix of human bone and retain their natural trabecular structure. The mass of bone matrix in the tubes was chosen to reflect the range of trabecular bone mineral density values occurring in healthy subjects 35 and disease states (BMD values were 0, 50, 100, and 150 mg/cm^3^). Phantoms were constructed in 5 mL scintillation vials. To ensure homogeneity, the bone granules were first thoroughly mixed with oil before sequential addition of SDS and heated agar. Final mixtures of fat, bone, and agar were then continually agitated with a high‐speed touch‐mixer (Vortex‐Genie 2, Cole‐Parmer, London, UK) until solid. Again, all tubes formed a solid gel. The volume of the bone granules was much smaller than the volume of fluid in each vial (i.e., unmeasurable); the volume of the bone granules therefore was neglected in calculations of BMD.

The arrangement of the fat–water–bone phantom is shown in Figure 1c; microscopic images of fat–water emulsions containing trabecular bone are shown in Figure 1d.

**Figure 2:**
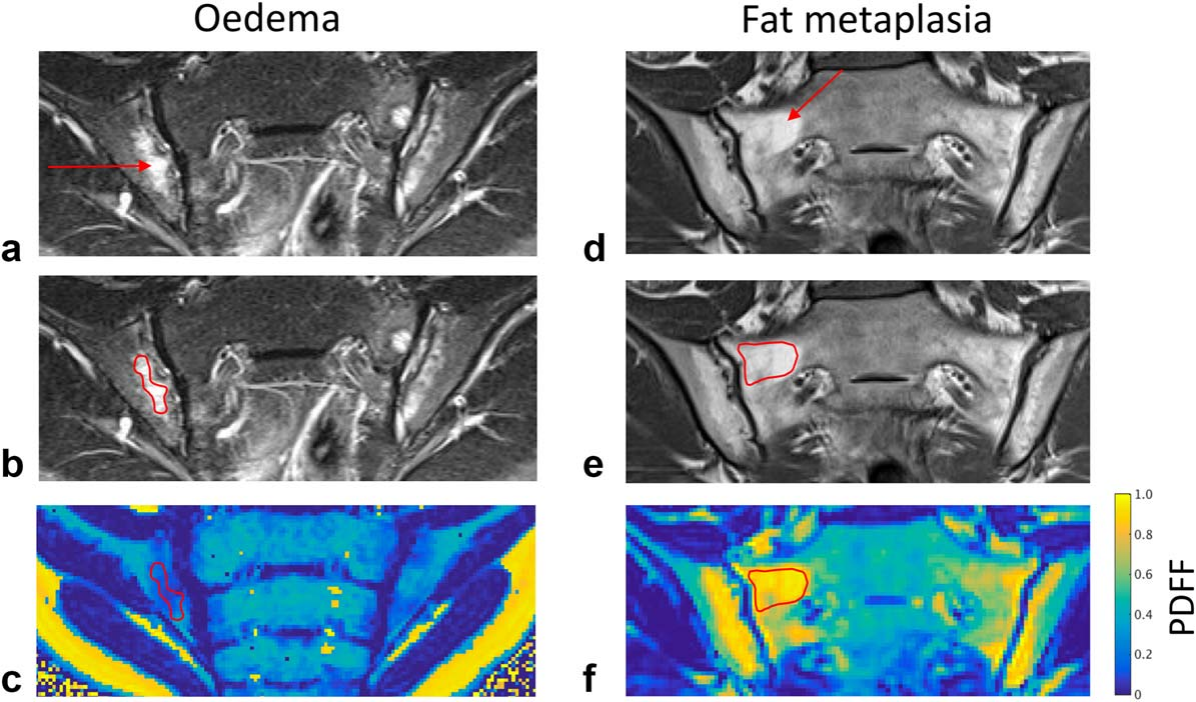
Delineation of edema and fat metaplasia. Areas of bone marrow edema and fat metaplasia were identified on T2‐weighted STIR (**a**) and T1‐weighted (**d**) spin echo images, respectively. Freehand regions of interest were placed on these images (**b**, **e**) and directly transferred to the corresponding anatomical location on PDFF maps (**c**, **f**) using the sacroiliac joints and sacral foraminae as anatomical landmarks. PDFF, proton density fat fraction; STIR, short tau inversion recovery.

### Patients and Volunteers

Subjects were prospectively recruited by rheumatologists working in a specialist tertiary rheumatology clinic. Patients aged between 12 and 30 years were included in the study if they had a known diagnosis of sacroiliitis (diagnosed previously on MRI) or were suspected of having sacroiliitis after assessment by a rheumatologist. All patients with sacroiliitis fulfilled diagnostic criteria for enthesitis‐related arthritis (with axial involvement) or nonradiographic axial spondyloarthritis 36, 37, 38. Patients with suspected sacroiliitis at the time of the scan were subsequently treated as controls if the clinical MRI scan and clinical assessment were normal. In addition, eight healthy volunteers aged between 18 and 30 years had repeat MRI scans of the sacroiliac joints 1 month apart to assess measurement repeatability.

### Imaging Sequence and Reconstruction

CSE‐MRI of phantoms and all subjects was performed on a wide‐bore 3.0T clinical system (Ingenia, Philips, Amsterdam, Netherlands), with integrated posterior and anterior surface coils using a 3D spoiled gradient echo recalled acquisition. Images of patients and volunteers were acquired in a tilted coronal plane parallel to the long axis of the sacrum, and 40 slices were acquired with a slice thickness of 2 mm. Imaging was performed using a vendor‐supplied gradient echo sequence (Philips mDixon Quant; Philips Healthcare, Andover, Massachusetts, USA), which acquires six evenly‐spaced echoes (first echo time 1.17 ms, echo spacing 1.6 ms) using bipolar readout gradients 39, 40, 41. PDFF and R2* maps were generated inline, assuming a 10‐peak model of human adipose tissue, as previously described 40, 42. Other sequences parameters included: time to repetition (TR) 25 ms, flip angle 3 °, matrix size 320 × 320, pixel spacing 1.76 mm × 1.76 mm, and pixel bandwidth 394 Hz/Px. Parallel imaging was not used. Additionally, all patients underwent a standard clinical MRI scan on the same day on a 1.5T system (Avanto, Siemens, Berlin, Germany) with coronal T_1_‐weighted and T_2_‐weighted STIR sequences 14, which were acquired in the same tilted coronal plane as the CSE scans.

### Statistical Analysis

For the phantom experiments, statistical analyses were based on those used in previous phantom studies using fat–water phantoms 26. PDFF and R2* measurements were performed using circular regions‐of‐interest (ROIs) placed on axial slices through the phantom tubes, taking care to avoid the edges of the emulsions. ROIs were placed in identical locations on PDFF and R2* maps. To assess the accuracy of FF measurements, linear regression was performed between known and measured FFs, and two‐sided *t* tests were used to determine whether there were statistically significant differences between obtained slope values and 1.0, and obtained intercept values and 0.0 (∝ = 0.05). To assess the relationship between BMD and R2*, linear regression was performed between measured R2* values and known BMD values, and analysis of covariance was used to determine whether obtained slope and intercept values were significantly different in vials containing fat compared to 0% fat‐fraction vials.

For the patient study, areas of bone marrow edema and/or fat metaplasia in patients with sacroiliitis were identified on T_2_‐weighted STIR and T_1_‐weighted images, respectively, and freehand ROIs were placed on those areas—taking care to avoid normal bone—by a radiology registrar (t.j.p.b.) with 4 years of experience in musculoskeletal MRI (see Fig. 2). A maximum of four ROIs for bone marrow edema and four ROIs for fat metaplasia were used in each subject. Using the synovial and ligamentous portions of the sacroiliac joints and sacral foramina as fixed anatomical landmarks, ROIs were then transferred manually onto PDFF maps at the same anatomical location, and mean PDFF and R2* values were recorded for each ROI. In controls with mechanical back pain, freehand ROIs were placed on the largest possible areas of subchondral bone marrow on both the sacral and ilial sides of the joint for both the left and right sacroiliac joints on a single slice. To determine whether there were statistically significant differences in PDFF and R2* between areas of normal bone marrow, edema, and fat metaplasia, data were analyzed using a multilevel mixed‐effects linear regression model on Stata 14.1 (StataCorp, College Station, Texas, USA), which accounted for repeated observations in individual patients. Tissue type (i.e., normal marrow, edema, or fat metaplasia) was used as a predictor variable; PDFF or R2* were used as outcome variables; and subject number was included as a grouping variable.

To assess the repeatability of PDFF and R2* measurements, four ROIs (one ROI for each side of the joint, for both left and right sacroiliac joints) were placed on the subchondral bone using a single representative slice through the sacroiliac joints. ROI data were analyzed using Bland‐Altman 95% limits of agreement, with adjustment of the limits to account for multiple observations for each individual 43, as well as using the intraclass correlation coefficient.

## RESULTS

### Fat–Water and Fat–Water–Bone Phantoms

**Figure 3:**
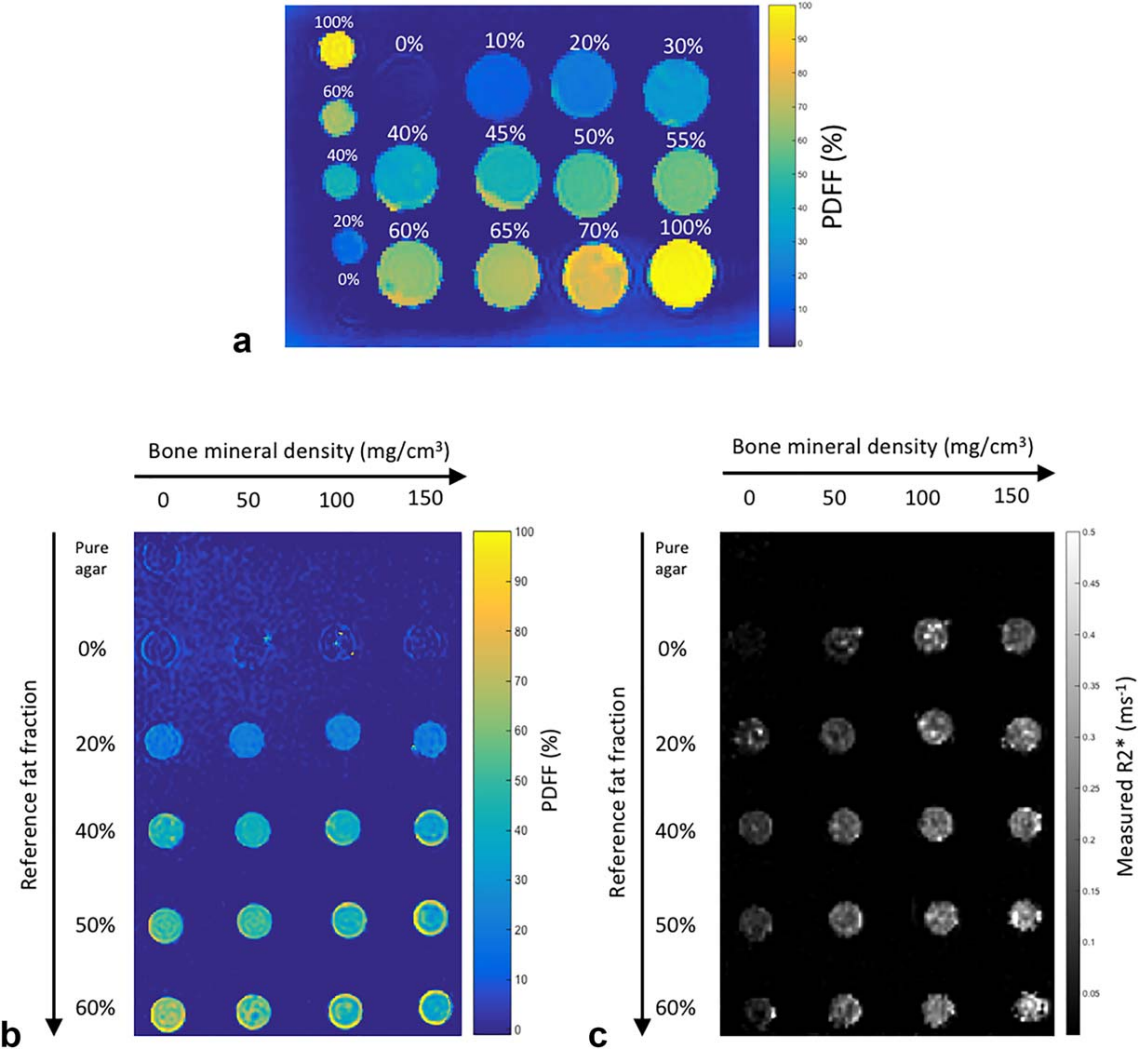
Fat–water and fat–water–bone phantoms. (**a**) PDFF maps from the fat–water phantom. Labels indicate reference FF values, which vary between 0 and 100%. (**b** and (**c**) PDFF and R2* maps from the fat–water–bone phantom. Reference FF values vary by row, whereas bone mineral density values vary by column. FF, fat fraction; PDFF, proton density fat fraction.

PDFF maps from the fat–water phantom are shown in Figure 3a. PDFF and R2* maps from the fat–water–bone phantom are shown in Figures 3b and 3c.

**Figure 4:**
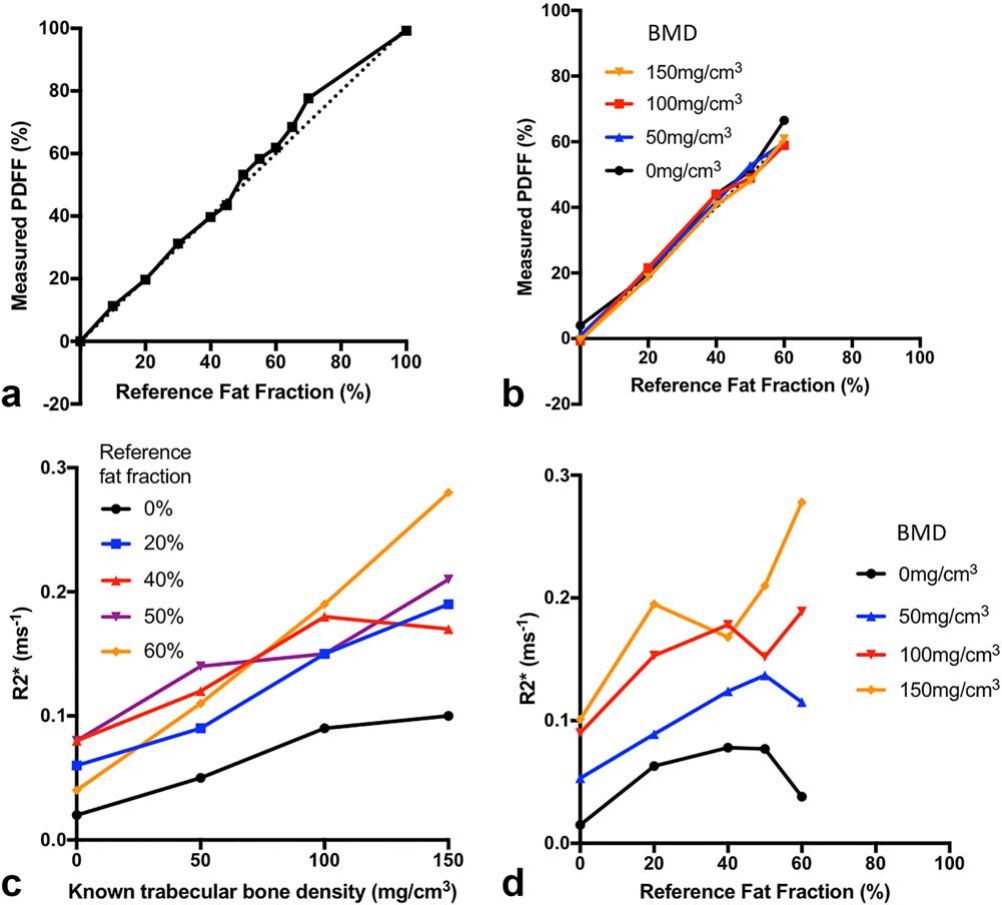
Relationship of PDFF and R2* with reference FF and BMD. (**a**) PDFF measurements agree closely with reference FFs in the absence of bone. (**b**) Increases in bone density have minimal impact on PDFF measurements over a physiological BMD range. (**c**) R2* measurements are linearly related to BMD, although the slope varies depending on fat content. (**d**) Variations in FF also influence the measured R2* for a given BMD. BMD, bone mineral density; FF, fat fraction; PDFF, proton density fat fraction.

Figures 4a and 4b show the relationship between reference FF and measured PDFF values in the fat–water and fat–water–bone phantoms. In the absence of bone (Fig. 2a), measured PDFF values agreed closely with reference FF values (slope = 1.03 ± 0.027, intercept = 0.0036 ± 0.014). Despite the presence of trabecular bone, measured PDFF values also agreed closely with reference FF values in the fat–water–bone phantom (Fig. 4b). Results of the linear regression analysis are given in Table 1a.

**Table 1:** Results of Linear Regression Analysis.

A. Measured PDFF Versus Reference Fat Fraction				
BMD	0 mg/cm^3^	50 mg/cm^3^	100 mg/cm^3^	150 mg/cm^3^
R^2^	0.989	0.998	0.991	0.998
Slope	1.03 ± 0.062	1.00 ± 0.026	0.99 ± 0.053	1.01 ± 0.029
	*P* = 0.59	*P* = 0.90	*P* = 0.85	*P* = 0.71
Intercept	0.018 ± 0.025	0.010 ± 0.011	0.0090 ± 0.021	−0.008 ± 0.012
	*P* = 0.47	*P* = 0.34	*P* = 0.68	*P* = 0.49

B. Measured R2* Versus Known BMD					
Reference FF	0%	20%	40%	50%	60%
R^2^	0.956	0.985	0.841	0.941	0.997
Slope	0.00056 ± 8.5x10^−5^	0.00090 ± 7.7 × 10^−5^	0.00066 ± 0.00020	0.00080 ± 0.00014	0.0016 ± 6.3 × 10^−5^
	Baseline	*P* = 0.39	*P* = 0.99	*P* = 0.67	*P* = 0.002
Intercept	0.023 ± 0.0079	0.055 ± 0.0072	0.088 ± 0.019	0.085 ± 0.013	0.035 ± 0.0059
	Baseline	*P* = 0.27	*P* = 0.014	*P* = 0.026	*P* = 0.88

**A.** Measured PDFF values agreed closely with known FFs for all BMD values in the phantom.

**B.** Measured R2* values were linearly related to BMD (all slope values were significantly greater than 0), although the slope varied significantly depending on the FF. *P*‐values relate to comparison with the 0% FF vial using analysis of covariance.

BMD, bone mineral density; FF, fat fraction; PDFF, proton density fat fraction.

Figure 4c shows the relationship between trabecular BMD and measured R2* values, and results of the linear regression analysis are given in Table 1b. In the absence of fat, there was a significant linear relationship between trabecular BMD and R2*. Linear relationships also were observed in vials containing fat, although slope values were increased compared to the 0% FF vial. Comparison of slope values between vials containing fat (i.e., between 20%, 40%, 50%, and 60% FFs) revealed no significant differences between FFs of 20%, 40%, and 50%; however, slope values were significantly increased at 60% FF. Figure 4d shows the relationship between R2* and reference FF. Although the nature of this relationship was complex, increases in FF at higher bone concentrations tended to increase R2* measurements.

### Patients and Volunteers

For the patient study, 18 subjects (12 males and 6 females) were prospectively recruited with a mean age of 17 y 5 m ± 2 y 11 m (mean ± standard deviation). Of these, eight subjects (6 males and 2 females, with mean age 18 y 2 m ± 3 y 4 m) had evidence of active or chronic sacroiliitis using conventional MRI. The remaining 10 subjects (6 males and 4 females, with mean age 16 y 11 m ± 2 y 6 m) had normal conventional MRI scans and no clinical or biochemical evidence of sacroiliitis, and therefore were treated as controls. Images from all subjects were of high quality; there were minor artifacts at the edges of the field of view in some subjects, but these did not overlap the pelvis or sacroiliac joints.

**Figure 5:**
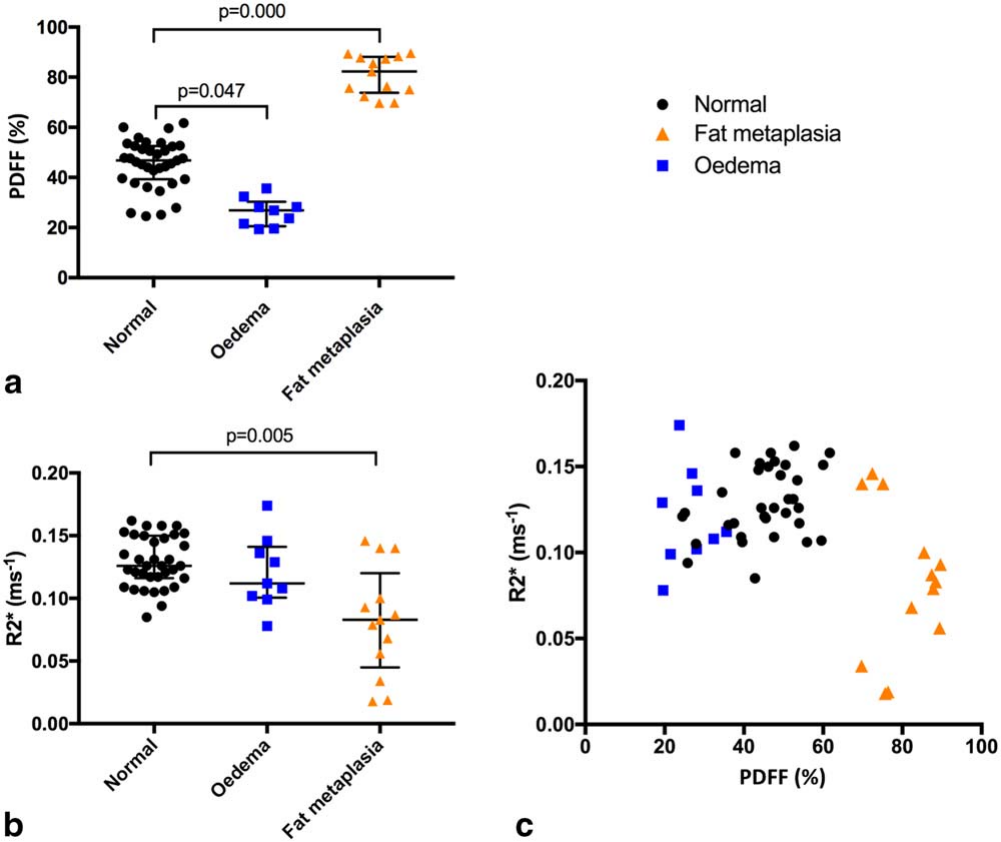
PDFF and R2* values in areas of normal bone marrow, bone marrow edema, and fat metaplasia. (**a**) PDFF values were significantly increased in areas of fat metaplasia, and significantly reduced in areas of bone marrow edema compared to normal subchondral marrow. (**b**) R2* values were significantly reduced in areas of fat metaplasia but not significantly altered in areas of edema. (**c**) PDFF and R2* measurements are shown on a scatterplot; areas of fat metaplasia exhibit both increases in PDFF and reductions in R2*. BMD, bone mineral density; FF, fat fraction; PDFF, proton density fat fraction.

**Figure 6:**
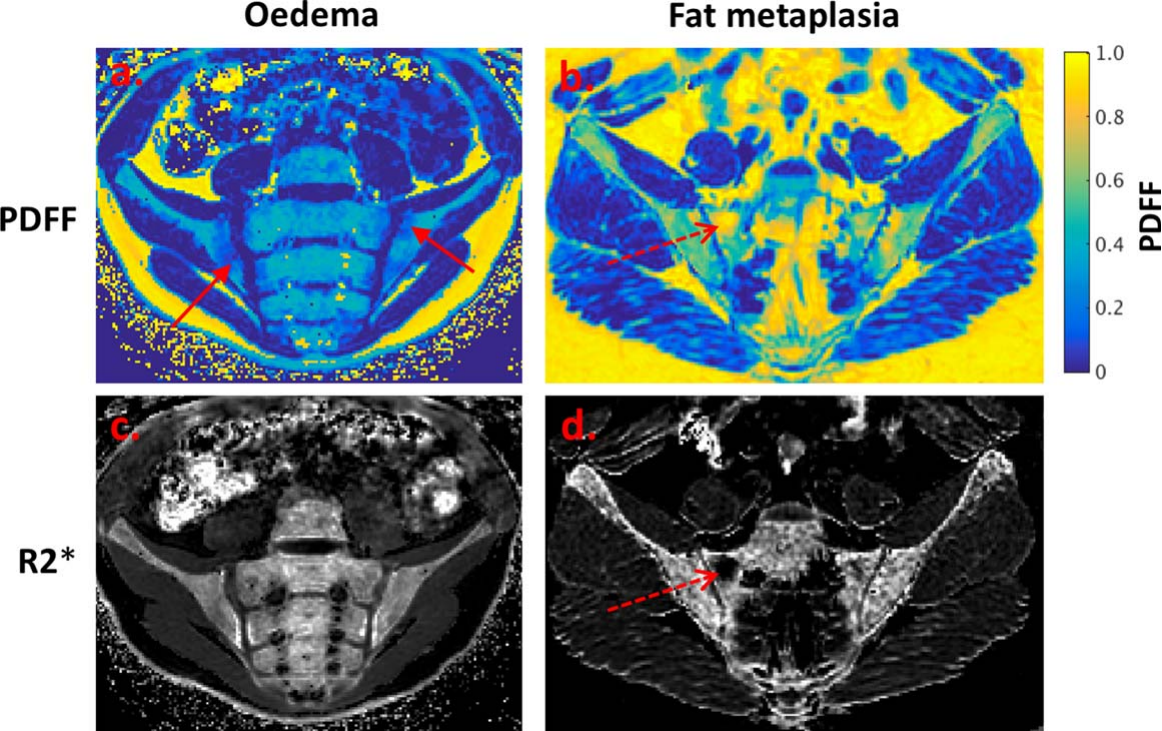
PDFF maps (**a**, **b**) and R2* maps (**c**, **d**) demonstrating bone marrow edema and fat metaplasia in patients with sacroiliitis. Bone marrow edema (**a**, **c**; solid red arrow) causes a decrease in PDFF but no change in R2*. Fat metaplasia (**b**, **d**; dashed red arrow) causes an increase in PDFF and a reduction in R2*. The reduction in R2* may indicate a loss of bone mineral density. PDFF, proton density fat fraction.

PDFF and R2* measurements are compared in normal marrow, marrow edema, and fat metaplasia in Figure 5. Example PDFF and R2* maps in patients with active and chronic sacroiliitis are shown in Figure 6. Median (interquartile range (IQR)) PDFF values were 46.8% (39.5%–52.5%) in normal bone marrow, 26.9% (21.5–28.2%) in bone marrow edema, and 82.3% (75.1%–87.8%) in fat metaplasia. Compared to normal marrow, PDFF measurements were significantly lower in areas of edema (*P* = 0.047), and were significantly higher in areas of fat metaplasia (*P* = 0.000). Median (IQR) R2* values were 0.126 ms^−1^ (0.117–0.149 ms^−1^) in normal marrow, 0.112 ms^−1^ (0.102–0136 ms^−1^) in bone marrow edema, and 0.083 ms^−1^ (0.056–0.100 ms^−1^) in fat metaplasia. R2* was significantly lower in areas of fat metaplasia (*P* = 0.005), but there was no significant difference in R2* between normal bone and bone marrow edema (*P* = 0.461).

### Repeatability

**Figure 7:**
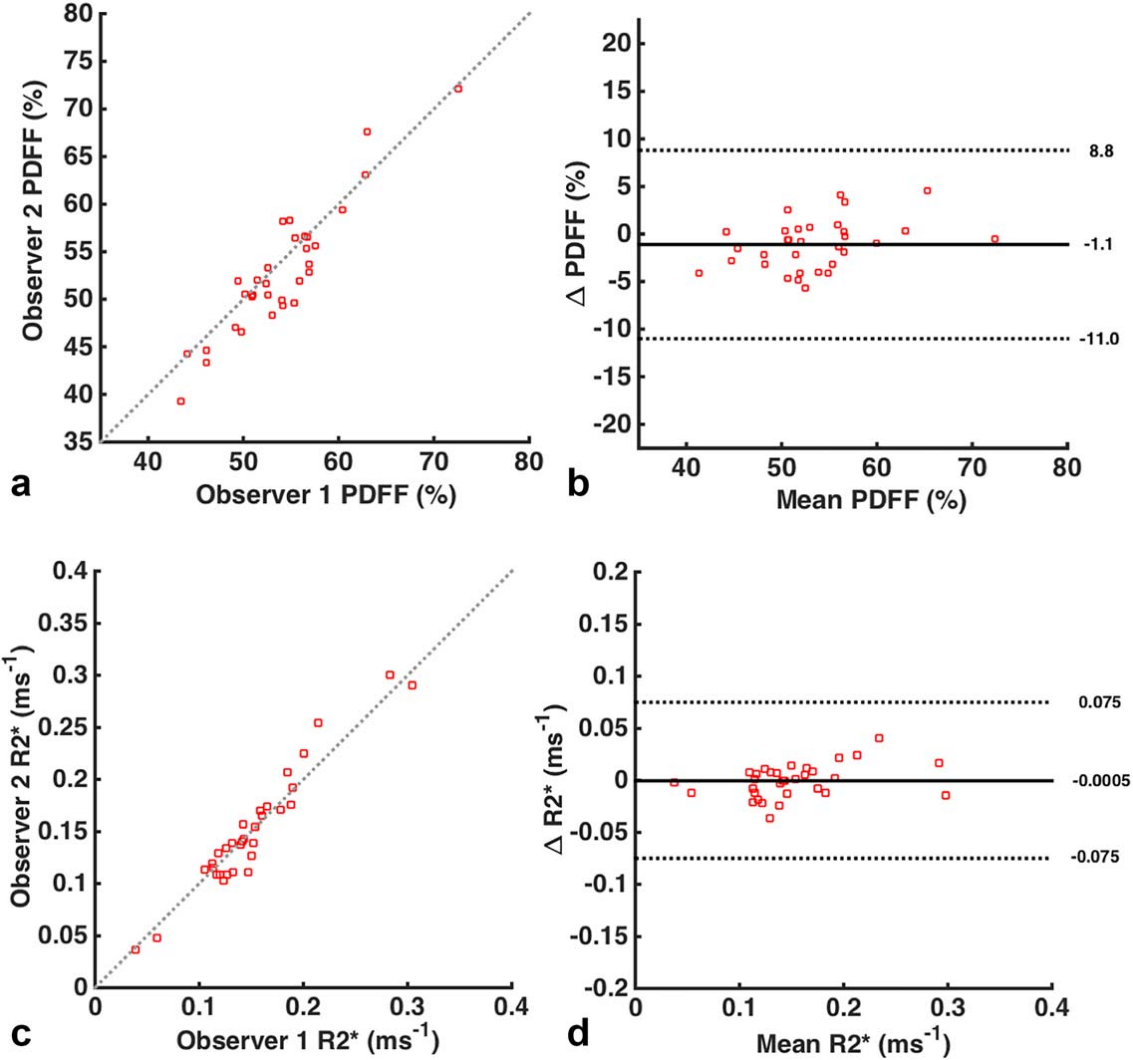
Bland‐Altman plots. The figures demonstrate the repeatability of observations for PDFF (**a**, **b**) and R2* (**c**, **d**) measurements, using scatterplots (**a**, **c**) and Bland‐Altman 95% limits of agreement (**b**, **d**). Limits were adjusted to account for the presence of multiple observations for each individual. PDFF, proton density fat fraction.

For repeatability assessment, eight healthy volunteers (seven males and one female, mean age 31 y 0 m ± 3 y 5 m) were recruited and scanned on two occasions 1 month apart. After adjusting the limits to account for multiple observations per individual, Bland Altman 95% limits of agreement were −1.1 ± 9.9% for PDFF and −0.0005 ± 0.075 ms^−1^ for R2* (Fig. 7). Intraclass correlation coefficients were 0.87 for PDFF and 0.98 for R2*.

## DISCUSSION

Here, we present a new approach to the assessment of active and chronic joint inflammation in spondyloarthritis using CSE‐MRI. We show that PDFF measurements accurately reflect changes in bone marrow composition in areas of edema and fat metaplasia, which can be viewed as active inflammatory and structural lesions, respectively. Additionally, we show that R2* measurements are reduced in areas of fat metaplasia, which may be due to a reduction in bone mineral density. Inflammation and structural damage are hallmarks of spondyloarthritis, and CSE‐MRI could have a multitude of clinical and research applications in this disease, including monitoring inflammatory activity and structural damage over time. Although we focused on spondyloarthritis in this study, it is anticipated that quantitative CSE‐MRI could find utility in other diseases affecting the bone marrow, such as multiple myeloma or osteomyelitis.

In our fat–water and fat–water–bone phantom experiments, PDFF measurements agreed closely with reference FF values over a physiologically relevant range, and essentially were unaffected by variations in BMD. In patients, PDFF measurements were significantly decreased in areas of bone marrow edema and were significantly increased in areas of fat metaplasia, suggesting that PDFF is altered at both actively and chronically inflamed sites. Decreases in PDFF at edematous sites likely are due to the presence of an inflammatory exudate in the juxta‐articular marrow 44, whereas increases in fat content may be due to increases in the proportion or size of adipocytes in the marrow. Given that fat metaplasia is known to be preceded by inflammation 12, 15, our data imply that the bone marrow undergoes biphasic changes in fat content with inflammation, which can be directly measured using CSE‐MRI.

Our phantom experiments also indicated a positive association between R2* and BMD 23, 45. A number of authors have previously explored the use of R2* or R2' as markers of osteoporosis, and these parameters have been found to parallel apparent density measured by dual‐energy X‐ray absorptiometry (DEXA) or quantitative CT (QCT) 46, 47, 48, 49, 50. However, our results indicate that R2* measurements also are influenced by PDFF and cannot be regarded as a pure marker of BMD when fat content varies as well. Importantly, R2* values also are influenced by trabecular geometry 51, the assumption of a single system R2* decay term for the marrow 30, and the arrangement of the fat and water compartments 52. For example, in patients with muscular dystrophy, the highest R2* values occur in muscles where fat and normal muscle are interdigitated, whereas R2* measurements in patients with heavily fat‐infiltrated muscles are comparable to those seen in normal subjects 52. Nonetheless, our results are somewhat different to those observed in patients with muscular dystrophy in that areas of bone marrow edema had lower PDFF values than normal marrow but did not undergo a significant reduction in R2*. Conversely, there was a significant reduction in R2* in areas of fat metaplasia. Given the known relationship between R2* and BMD, this result may be due to a reduction in trabecular bone mineral density. Previous studies also support this suggestion. For example, Kühn et al. showed that R2* measurements could discriminate normal and osteoporotic patients with reasonably high levels of sensitivity and specificity despite concomitant variations in fat content in osteoporotic individuals 46.

From a biological perspective, reductions in BMD in areas of fat metaplasia might be expected because inflammatory cytokines such as tumor necrosis factor alpha are known to increase osteoclastic activity 4, 53, 54. Additionally, increases in fat content and reduced bone mineral density in areas of fat metaplasia could occur through a common mechanism. Previous studies suggest differentiation of marrow stem cells into adipocytes occurs at the expense of osteoblasts 55, and it may be that this balance has been perturbed by previous inflammation.

There is an extensive literature on the use of qualitative scoring systems to evaluate inflammation of the sacroiliac joints 11, 12, 14 but relatively few studies using quantitative MRI for this purpose. Several small studies have demonstrated that diffusion‐weighted MRI can be used to quantify inflammation in both adults and children 56, 57, 58, 59, but problems with the reproducibility of this technique across different scanning platforms and sites may limit its widespread application 60, 61. There has been very little previous research using CSE‐MRI to evaluate marrow inflammation. A conference abstract by Lee et al. briefly describes the use of three‐point Dixon MRI to measure changes in FF at vertebral corners in spondyloarthritis 62, but this work did not utilize R2* correction or measurement, did not differentiate between FF changes occurring in acute and chronic inflammation, and did not involve specific evaluation of the sacroiliac joints.

As a method for quantifying joint inflammation, the proposed method has a number of advantages over existing approaches. Firstly, the repeatability of PDFF and R2* measurements was excellent, and differences between repeated measurements were much smaller than differences between normal bone, edema, and fat metaplasia. Intraclass correlation values were similar to previously quoted values for visual scoring 12. Secondly, the volumetric nature of the acquisition means that images can be reformatted in multiple planes without the need for repeated acquisitions; using conventional MRI, repeated images are typically acquired in axial, coronal, and sometimes sagittal planes as well 14. Therefore, CSE‐MRI may help reduce scan time and could easily be applied to whole body imaging, In addition, whole body MRI can be used to identify occult inflammation and facilitate early diagnosis 18, 19, and the method proposed here is likely to be much faster than existing spin echo‐based approaches. Even greater acceleration could be achieved using compressed sensing and parallel imaging techniques, as recently described 63. CSE‐MRI also might be used to generate whole body R2* maps, which could be applied to monitor structural damage and progression throughout the spine.

The fat–water–bone phantoms described here provide a potential framework for refining PDFF and R2* measurements and for evaluating the accuracy of individual CSE‐MRI methods in the bone marrow. Although other authors have described phantoms in which fat–water mixtures are used to fill cadaveric human bone 21, the approach described here allows for direct manipulation of trabecular bone mineral density using standardized bone granules and could be replicated easily at other sites. Fat–water–bone phantoms may be useful because differences in hardware, sequences, and postprocessing techniques between imaging platforms could all potentially influence PDFF and R2* measurements. The optimal method is likely to depend on the specific sequence, imaging platform, image reconstruction software, and anatomical region. Ideally, it would be possible to compare PDFF and R2* measurements across multiple sites; further research is required to establish the optimal imaging method on different platforms and to establish the reproducibility of these measurements.

A limitation of this study is that the analyses used here assumed a multipeak fat spectrum that is based on measurements from human adipose tissue rather than the bone marrow. However, previous studies suggest that spectra from bone marrow and human adipose tissue are extremely similar 42. Additionally, the reference FF values described in this study do not represent true gold standard PDFF measurements, which ideally would be performed using spectroscopy. Nonetheless, reference FFs and true PDFF values are likely to agree relatively closely, and the conclusions drawn here are not contingent on precise assessment of accuracy in phantoms. It is unclear whether the fat spectrum in areas of fat metaplasia is altered compared to normal marrow: accurate characterization of the fat spectrum in areas of fat metaplasia (e.g., using spectroscopy) would help to clarify this issue. Accurate spectral fat modelling also may help reduce confounding effects on R2* measurements 25. Finally, variations in temperature alter the magnitude of chemical shift between fat and water 64, which was not accounted for in this study. This may have a small impact on the phantom experiments, which were performed at room temperature rather than at body temperature.

The patients included in this study represent a relatively young spondyloarthritis cohort; most subjects were in their teens. Although imaging may be particularly valuable in this early‐onset group 9, changes in skeletal maturity may influence PDFF and R2* measurements and therefore make quantification of inflammation more difficult. Younger patients may have more cellular (i.e., less fatty) marrow and unossified juxta‐articular bone, which may mimic bone marrow edema 65. Maturity‐related changes in marrow composition potentially could be adjusted for using background marrow as a reference. It also is possible that areas of immature bone could be differentiated based on their R2* measurements, which would be expected to be lower than those in fully ossified mature bone. Ultimately, it would be desirable to develop a technique which could be used to quantify inflammation across the full age spectrum.

A specific limitation of the fat–water–bone phantom is that the phantoms were not perfectly homogenous at high FF and BMD values. PDFF values appeared slightly higher at the edges of the emulsion, which may be due to some separation of the oil from the gel. The impact on absolute PDFF measurements for each vial appeared to be minimal, although it is likely that this inhomogeneity had some influence on the measured R2* values, and it may have contributed to the complex dependence of R2* on FF. Using either solid animal fats or thick liquid phantoms, for example, containing carrageenan 31, may enable construction of more homogenous phantoms with high FFs. In the future, it would be useful to directly evaluate the relationship between BMD and R2* in vivo using DEXA or QCT, although this approach would be subject to ethical constraints given the use of ionizing radiation in a cohort of pediatric and young adult patients.

It should be noted that the bone granules used in this study only contain the mineral content of trabecular bone, meaning that the organic matrix is missing and any effect of collagen on the measured PDFF will not be present. Experiments with ex‐vivo human cortical bone samples demonstrate that collagen‐bound water makes up a significant fraction (approximately one‐third) of the water content of trabecular bone 66. At the echo times used for the CSE‐MRI in this work, the signal from this bound‐water fraction therefore will be absent due to its very short T2*. Karampinos et al. have demonstrated that this can result in overestimation of PDFF in comparison to spectroscopic methods that account for short T2* water species 67. In the future, using more sophisticated phantoms, including collagen‐bound water, may help overcome this bias.

## CONCLUSION

We have shown that PDFF measurements reflect changes in marrow composition in areas of bone marrow edema and fat metaplasia and could therefore be used to monitor both active inflammation and structural damage in spondyloarthritis. R2* measurements may provide additional information about marrow structure but also are influenced by fat content. The reduction in R2* in areas of fat metaplasia is a new finding and may be due to the loss of trabecular bone mineral density. Furthermore, we have described a series of fat–water and fat–water–bone phantoms that provide a potential framework for refinement of PDFF and R2* measurements and evaluation of the accuracy of individual CSE‐MRI methods in the bone marrow.
